# Cortical Thickness Estimation in Individuals With Cerebral Small Vessel Disease, Focal Atrophy, and Chronic Stroke Lesions

**DOI:** 10.3389/fnins.2020.598868

**Published:** 2020-12-14

**Authors:** Miracle Ozzoude, Joel Ramirez, Pradeep Reddy Raamana, Melissa F. Holmes, Kirstin Walker, Christopher J. M. Scott, Fuqiang Gao, Maged Goubran, Donna Kwan, Maria C. Tartaglia, Derek Beaton, Gustavo Saposnik, Ayman Hassan, Jane Lawrence-Dewar, Dariush Dowlatshahi, Stephen C. Strother, Sean Symons, Robert Bartha, Richard H. Swartz, Sandra E. Black

**Affiliations:** ^1^LC Campbell Cognitive Neurology Research, Hurvitz Brain Sciences Program, Sunnybrook Research Institute, University of Toronto, Toronto, ON, Canada; ^2^Rotman Research Institute, Baycrest Health Sciences, Toronto, ON, Canada; ^3^Department of Medical Biophysics, University of Toronto, Toronto, ON, Canada; ^4^Centre for Neuroscience Studies, Queens University, Kingston, ON, Canada; ^5^Tanz Centre for Research in Neurodegenerative Diseases, University of Toronto, Toronto, ON, Canada; ^6^Division of Neurology, Toronto Western Hospital, University Health Network, Toronto, ON, Canada; ^7^Stroke Outcomes and Decision Neuroscience Research Unit, Division of Neurology, St. Michael’s Hospital, University of Toronto, Toronto, ON, Canada; ^8^Thunder Bay Regional Health Research Institute, Thunder Bay, ON, Canada; ^9^Department of Medicine (Neurology), Ottawa Hospital Research Institute, University of Ottawa, Ottawa, ON, Canada; ^10^Department of Medical Imaging, Sunnybrook Health Sciences Centre, University of Toronto, Toronto, ON, Canada; ^11^Centre for Functional and Metabolic Mapping, Department of Medical Biophysics, Robarts Research Institute, University of Western Ontario, London, ON, Canada; ^12^Department of Medicine (Neurology), Sunnybrook Health Sciences Centre, University of Toronto, Toronto, ON, Canada

**Keywords:** cortical thickness, cerebrovascular disease, stroke, cerebral small vessel disease, FreeSurfer, ONDRI, MRI

## Abstract

**Background:**

Regional changes to cortical thickness in individuals with neurodegenerative and cerebrovascular diseases (CVD) can be estimated using specialized neuroimaging software. However, the presence of cerebral small vessel disease, focal atrophy, and cortico-subcortical stroke lesions, pose significant challenges that increase the likelihood of misclassification errors and segmentation failures.

**Purpose:**

The main goal of this study was to examine a correction procedure developed for enhancing FreeSurfer’s (FS’s) cortical thickness estimation tool, particularly when applied to the most challenging MRI obtained from participants with chronic stroke and CVD, with varying degrees of neurovascular lesions and brain atrophy.

**Methods:**

In 155 CVD participants enrolled in the Ontario Neurodegenerative Disease Research Initiative (ONDRI), FS outputs were compared between a fully automated, unmodified procedure and a corrected procedure that accounted for potential sources of error due to atrophy and neurovascular lesions. Quality control (QC) measures were obtained from both procedures. Association between cortical thickness and global cognitive status as assessed by the Montreal Cognitive Assessment (MoCA) score was also investigated from both procedures.

**Results:**

Corrected procedures increased “Acceptable” QC ratings from 18 to 76% for the cortical ribbon and from 38 to 92% for tissue segmentation. Corrected procedures reduced “Fail” ratings from 11 to 0% for the cortical ribbon and 62 to 8% for tissue segmentation. FS-based segmentation of T1-weighted white matter hypointensities were significantly greater in the corrected procedure (5.8 mL vs. 15.9 mL, *p* < 0.001). The unmodified procedure yielded no significant associations with global cognitive status, whereas the corrected procedure yielded positive associations between MoCA total score and clusters of cortical thickness in the left superior parietal (*p* = 0.018) and left insula (*p* = 0.04) regions. Further analyses with the corrected cortical thickness results and MoCA subscores showed a positive association between left superior parietal cortical thickness and Attention (*p* < 0.001).

**Conclusion:**

These findings suggest that correction procedures which account for brain atrophy and neurovascular lesions can significantly improve FS’s segmentation results and reduce failure rates, thus maximizing power by preventing the loss of our important study participants. Future work will examine relationships between cortical thickness, cerebral small vessel disease, and cognitive dysfunction due to neurodegenerative disease in the ONDRI study.

## Introduction

Cortical thickness quantification obtained from magnetic resonance imaging (MRI) can be used to examine regional variations of the cerebral cortex that have been associated with normal aging and dementia due to neurodegeneration (Fischl and Dale, 2000; Salat et al., 2004; Lerch and Evans, 2005; Raamana et al., 2015; Redolfi et al., 2015). Cortical thinning in specific topographical regions of the brain has been used to accurately determine patterns of neurodegeneration in mild cognitive impairment (MCI) (Raamana et al., 2014), Alzheimer’s disease (AD), frontotemporal dementia (FTD) (Du et al., 2007; Lerch et al., 2008; Bakkour et al., 2009; Richards et al., 2009; Cho et al., 2012; Hartikainen et al., 2012; Paternicó et al., 2016; Vuksanović et al., 2019), Parkinson’s disease (Uribe et al., 2016; Gao et al., 2018; Yau et al., 2018; Wilson et al., 2019), amyotrophic lateral sclerosis (ALS) (Verstraete et al., 2012; Mezzapesa et al., 2013; Schuster et al., 2014; Walhout et al., 2015), and vascular cognitive impairment (Seo et al., 2010; Kim et al., 2014; Jung et al., 2018).

FreeSurfer (FS) is a neuroimaging software package that includes a widely used surface-based analysis technique that is able to automatically estimate cortical thickness from T1-weighted MRI (Fischl, 2012). However, degraded image quality and subtle changes introduced by pathology makes it challenging for FS to achieve accurate and reliable brain extraction and white matter (WM) segmentation (Gronenschild et al., 2012; Moore et al., 2012; Iscan et al., 2015; Li et al., 2015a; Backhausen et al., 2016; Schwarz et al., 2016). Although FS provides manual intervention steps to troubleshoot its output (e.g., via control points, WM lesion edits, and pial edits), they are labor-intensive. Further, they may introduce user-bias (Klapwijk et al., 2019), especially in MRI from individuals with significant brain atrophy, cortical stroke lesions, and cerebral small vessel disease. Previous studies examining FS manual correction approaches found that while manual editing may result in differences in morphometrical estimation between the methods in some brain regions (Li et al., 2015b; McCarthy et al., 2015; Canna et al., 2018; Guenette et al., 2018; Waters et al., 2019; Beelen et al., 2020), sensitivity to cognitive-behavioral changes are inconsistent at individual or clinical group levels (McCarthy et al., 2015; Guenette et al., 2018; Waters et al., 2019).

Estimation of cortical thickness in patients with cerebrovascular disease (CVD) can be the most challenging due to cortico-subcortical chronic stroke lesions, significant volumes of white matter hyperintensities (WMH), lacunar infarcts, MRI-visible perivascular spaces (PVS), cortical microinfarcts, and the presence of focal brain atrophy. Given that the performance of FS’s tissue classification is highly dependent on a uniform intensity of voxels in a particular brain region and the integrity of the neighboring voxels, vascular lesions and focal brain atrophy often result in erroneous tissue segmentations, particularly in regions with high surface area and curvature (Fischl et al., 2002). These challenges reduce the accuracy of tissue segmentation, which in turn reduces the accuracy of cortical thickness estimation. Since many age-related neurodegenerative diseases have focal and diffuse brain atrophy that is further exacerbated by comorbid cerebrovascular pathology (Schneider and Bennett, 2010; Khlif et al., 2019a, b), additional procedures to account for these potentially challenging variations in image contrast are needed.

In this paper, we examined results from a FS correction procedure that was applied to MRI obtained from a heterogeneous CVD cohort with varying degrees cerebral small vessel disease, chronic cortico-subcortical stroke lesions, and brain atrophy.

## Materials and Methods

### Study Participants

Participants (*N* = 155) recruited to the CVD cohort of the Ontario Neurodegenerative Disease Research Initiative (ONDRI) (Farhan et al., 2017)^1^ were selected for methodological validation of the FS correction procedure for cortical thickness estimation. The ONDRI study is a multi-modal, multi-site observational research study investigating individuals with neurodegenerative diseases. Study participants were recruited at various health centers across Ontario, Canada: London Health Science Centre and Parkwood Institute in London; Hamilton General Hospital and McMaster Medical Centre in Hamilton; The Ottawa Civic Hospital in Ottawa; Thunder Bay Regional Health Sciences Centre in Thunder Bay; St. Michael’s Hospital, Sunnybrook Health Sciences Centre, Baycrest Health Sciences, Centre for Addiction and Mental Health, and Toronto Western Hospital (University Health Network) in Toronto.

Detailed inclusion and exclusion criteria for the ONDRI CVD participants are previously reported (Farhan et al., 2017; Sunderland et al., 2020). Briefly, participants who had experienced a mild to moderate ischemic stroke event, documented with MRI or CT, over 3 months prior to enrollment, a Modified Rankin Scale (MRS) score (van Swieten et al., 1988) ranging from 0 to 3, and a Montreal Cognitive Assessment (MoCA) score (Nasreddine et al., 2005) ranging 18–30 were included. Participants were excluded if they had severe cognitive impairment, aphasia, a non-vascular cause of symptoms, inability to write or had severe functional disability preventing them to perform assessments, a history of dementia prior to the stroke event, had severe claustrophobia or other contra-indications to MRI procedures. Ethics approval was obtained from all participating institutions and performed in accordance with the Declaration of Helsinki. All participants provided informed consent, and subsequently underwent clinical evaluation, MRI, and other assessments as part of the full ONDRI protocol (Farhan et al., 2017).

### MRI Acquisition and Pre-processing

Magnetic resonance imaging protocols were harmonized with the Canadian Dementia Imaging Protocol (CDIP) (Duchesne et al., 2019), and were in compliance with the National Institute of Neurological Disorders and Stroke–Canadian Stroke Network Vascular Cognitive Impairment Harmonization Standards (Hachinski et al., 2006). Detailed MRI protocols are reported elsewhere (Haddad et al., 2020; Ramirez et al., 2020). In brief, the structural MRI used in the current study include: a high-resolution 3D T1-weighted (T1), an interleaved proton density (PD) and T2-weighted (T2), and a T2 fluid-attenuated inversion recovery (FLAIR) images.

Ontario Neurodegenerative Disease Research Initiative’s structural image processing pipeline (Ramirez et al., 2020) will be considered as the pre-processing step for the *Corrected FS* procedure. ONDRI’s neuroimaging platform used previously published and validated methods, where outputs were further subjected to comprehensive quality control (QC) measures from ONDRI’s neuroinformatic platform using a novel outlier detection algorithm for the identification of anomalous data (Beaton et al., 2019; Sunderland et al., 2019). Briefly, interleaved PD and T2 images and FLAIR images were co-registered to the T1 and a PD-T2 based intra-cranial vault mask was automatically generated and manually edited to create brain segmentation (skull stripping). Using this mask, the T1 was segmented using a multi-feature histogram method to generate a tissue segmentation containing gray matter, WM, and cerebrospinal fluid (CSF) volumes (Kovacevic et al., 2002), at which point ventricular CSF was manually relabeled and the cerebellum was manually removed from the image. WMH of presumed vascular origin and lacunes were automatically identified with FLAIR-based and PD-T2-based lesion segmentation pipelines, respectively and manual edits were applied to remove false positives and recover false negatives (Gibson et al., 2010; Ramirez et al., 2011). Furthermore, the PD-T2 lesion pipeline was used to capture enlarged PVS (Ramirez et al., 2011, 2015). Cortico-subcortical stroke lesions were identified and verified by an expert research neuroradiologist (FG) on T1 and FLAIR images, and manually traced by an experienced neuroimaging analyst (MH). The final output of the pipeline produced a skull-stripped brain mask with segmented voxels comprising of four different “normal tissue” classes and five different “lesion tissue” classes: normal appearing white matter (NAWM), normal appearing gray matter (NAGM), sulcal and ventricular cerebrospinal fluid (sCSF/vCSF), periventricular and deep WMH (pWMH/dWMH), lacunes, PVS, and cortico-subcortical stroke lesions. The skull stripped and lesion-labeled masks were introduced at different processing stages of the *Corrected FS* procedure described below.

### FreeSurfer (FS) Processing Overview

All scans were processed using the stable version of FS (Linux FSv6.0). Two methods were applied to the same participant’s MRI: (a) Unmodified FS and (b) Corrected FS. After applying the two methods, visual inspection was performed by two experienced neuroimaging analysts (MH = rater1; KW = rater2). Raters were blinded to the individual and segmentation method used. Discordant ratings were resolved by a research neuroradiologist (FG). The images were either rated a “pass” or “fail” based on the overall cortical ribbon and tissue segmentation as described in the “Quality Control Assessment Procedures” in the following section.

*Unmodified FS*: The unmodified procedure involved the standard reconstruction steps in the FS pipeline with the default settings on all participants without any manual interventions. Briefly, the standard reconstruction steps included skull stripping, WM segmentation, intensity normalization, surface reconstruction, subcortical segmentation, cortical parcellation and thickness (Fischl, 2012).

*Corrected FS*: The corrected procedure involved dividing the reconstruction steps into the following three stages in order to incorporate the skull stripped brain and lesion masks from the ONDRI processing pipeline into FS’s pipeline:

Stage 1 (autorecon1) This involved replacing the “skull stripped mask” (brainmask.mgz) generated by FS’s standard skull stripping method with an improved skull stripped mask from the ONDRI skull stripping method.

Stage 2 (autorecon2) The second intervention (-autorecon2) involved the integration of lesion masks from ONDRI into the initial version of brain tissue segmentation file (aseg.presurf.mgz) generated by FS’s standard segmentation of the brain which includes subcortical structures, WM, GM, CSF, and WM hypointensities. The lesions were given an index value of “77” corresponding to the lesion value in the FS pipeline.

Stage 3 Lastly, the modified aseg.presurf.mgz and brain mask were used as inputs in the last stage of FS pipeline stage 3 (-autorecon2-noaseg -autorecon3) for automatic cortical parcellation and statistics.

### Quality Control Assessment Procedures

**FIGURE 1:**
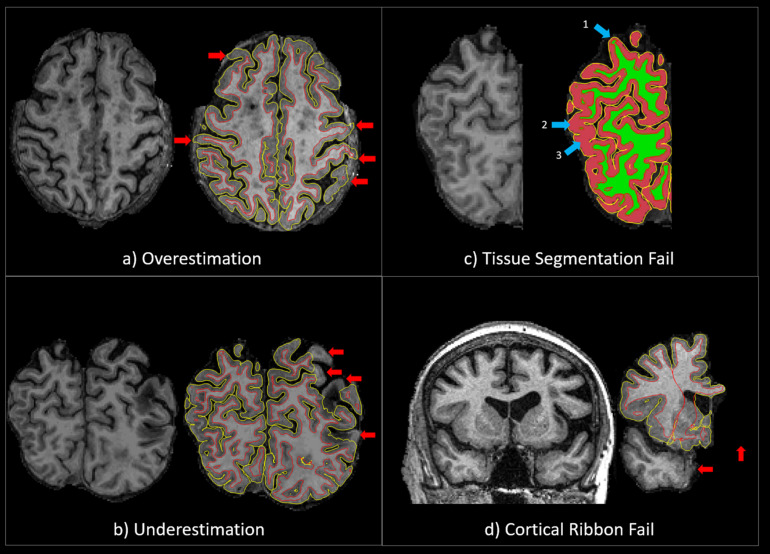
**(a)** An example of a QC “fail” rating due to significant overestimation of the cortical ribbon segmentation. The red arrows point to areas where the cortical ribbon segmentation erroneously classified significant areas of non-brain matter, such as the dura mater or skull, as cortex. **(b)** An example of a QC “fail” rating due to significant underestimation of the cortical ribbon segmentation. The red arrows point to areas where the cortical ribbon segmentation erroneously removed significant areas of the brain. **(c)** An example of a QC “fail” rating due to significant tissue segmentation errors. The 1st blue arrow points to an area where GM was misclassified as WM, and the 2nd and 3rd blue arrows point to areas WM was misclassified as GM. **(d)** An example of a QC “fail” rating due the complete absence of a cortical ribbon for an entire brain lobule or hemisphere. The red arrows point to areas of the brain (temporal lobe and one entire hemisphere) that have been erroneously removed.

The accuracy of the cortical ribbon and tissue segmentation from the Unmodified and Corrected FS procedures was evaluated using Freeview, a visualization tool that is packaged with FS. Using the T1 image as the reference, the cortical ribbon accuracy was assessed visually and given a “pass” or “fail” rating based on overestimation or underestimation. Overestimation was defined as a cortical ribbon segmentation that erroneously classified areas of non-brain matter, such as the dura mater or skull, as cortex. Underestimation was defined as a cortical ribbon segmentation that erroneously removed areas of the brain. A “pass” rating was given when the cortical ribbon segmentation showed minimal to no areas of over/underestimation. A “fail” rating was given when there were significant areas of overestimation and/or underestimation (Figures 1a,b), including the complete absence of a cortical ribbon for an entire brain lobule or hemisphere (Figure 1d). Additionally, using the T1 GM-WM intensity differences as a reference, the quality of the tissue segmentation (aseg.mgz) was given a pass/fail rating based on the accuracy of WM-GM boundary. For the tissue segmentation, a “pass” rating was given when the GM-WM boundary showed minimal to no errors in the GM-WM boundary. A “fail” rating was given when there were multiple brain regions where GM was misclassified as WM, and/or WM was misclassified as GM (Figure 1c). Two expert neuroimaging analysts performed the visual ratings and achieved strong inter-rater reliability results for the cortical ribbon segmentation (Cohen’s kappa (*k*) = 0.9, 95% C.I.: 0.7, 1.00, *p* < 0.001) and moderate inter-rater reliability results for the tissue segmentation (*k* = 0.7, 95% C.I.: 0.5, 0.9, *p* < 0.001) (McHugh, 2012).

### Statistical Analysis

Statistical analyses were conducted using Statistical Package for Social Sciences (SPSS v.24) and FS’s packaged analytic software when described. Paired sample *t*-tests were conducted to determine if the mean lesion volume was significantly different between the unmodified and corrected procedures. This was achieved using the “White matter Hypointensities” identified by FS, which was adjusted for head size using estimated total intracranial volume (eTIV) and log transformed.

A whole brain vertex-wise surface-based cortical thickness analysis was performed on both methods using the built-in general linear model (GLM). Thickness was calculated by the software as the distance between the GM and WM boundaries (also known as the pial surface boundaries) at every vertex in each hemisphere. Each participant’s cortex was anatomically parcellated with every sulcus and gyrus labeled, and resampled to FS’s default average surface map (fsaverage). A 10-mm full-width half-maximum (FWHM) Gaussian spatial smoothing kernel was applied to the surface to improve the signal-to-noise ratio. Age, stroke, and lacunar volumes were included as nuisance regressors. Stroke and lacunar volumes were head size corrected using eTIV.

Montreal Cognitive Assessment total score was included as a regressor of interest to determine the association between cortical thickness and global cognitive status in participants with CVD. Associations between cortical thickness and cognition were further explored using MoCA sub-scores (Visuospatial/Executive, Naming, Memory, Attention, Language, Abstraction, Delayed Recall, and Orientation). Monte Carlo simulations with 5000 iterations were used to correct for multiple comparisons. This method implemented a cluster-wise threshold of 2 (*p* = 0.01) and cluster-wise probability (*p*_(cwp)_) of *p* < 0.05 (two-sided). Bonferroni correction was applied across the two hemispheres.

## Results

Study participant demographics and clinical characteristics are summarized in Table 1. QC results are summarized in Table 2.

**TABLE 1:** Study participant demographics and neuroimaging volumetrics (*n* = 155).

*Demographics*	
Age (years)	69.35 (7.36)
Sex, n (%) female	49 (31.6)
Education, years	14.69 (2.88)
Modified Rankin Scale	1.01 (0.83)
Montreal Cognitive Assessment	25.29 (2.99)
***Neuroimaging Volumetrics, mm^3^***	
White matter hyperintensities	10167.5 (12837.2)
Lacunes	385.1 (766.7)
Enlarged perivascularspaces	80.0 (139.7)
Cortico-subcortical Stroke Lesions	6785.0 (17317.8)

*All data are presented as mean (SD) unless otherwise indicated. The data provided is based on the corrected FS procedure.*

**TABLE 2:** Quality control results for FreeSurfer Unmodified and Corrected procedures.

Description	Unmodified	Corrected
***Cortical Ribbon***		
Over-estimation	**26**%	**0**%
Under-estimation	**45**%	24%
Acceptable	**18**%	76%
Fail	**11**%	0%
***Tissue Segmentation***		
Pass	**38**%	92%
Fail	**62**%	8%

**FIGURE 2:**
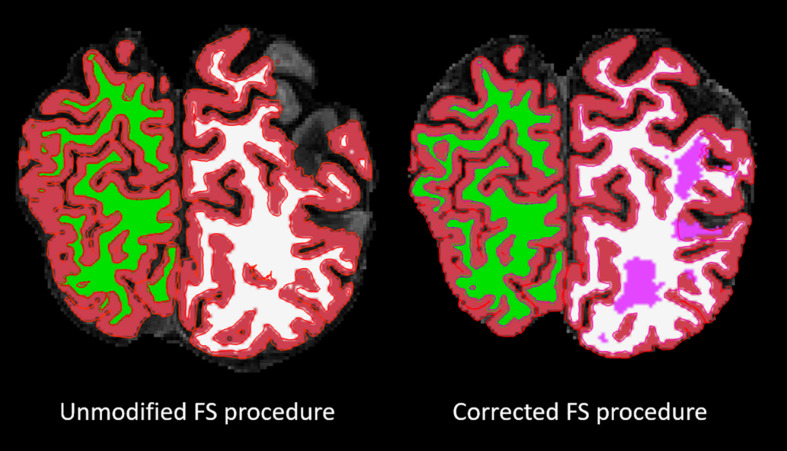
Overlaid on a skull-stripped T1, the left image shows the segmentation result from the FS unmodified procedure and the right image shows the segmentation result from the FS corrected procedure. The segmentation colors are as follows: red = GM; green/white = right/left WM; pink = lesion.

For the cortical ribbon QC, compared to the Unmodified FS procedure, the Corrected “Acceptable” ratings increased from 18 to 76%. For tissue segmentation QC, compared to the Unmodified FS procedure, the Corrected procedure’s “Acceptable” ratings increased from 38 to 92%. For the cortical ribbon QC, the “Fail” ratings were reduced from 11% (Unmodified) to 0% (Corrected). While for the tissue segmentation QC, the “Fail” ratings were reduced from 62 to 8% for Unmodified and Corrected procedures, respectively (e.g., Figure 2). Thus, for the subsequent brain-behavior analysis, we excluded 24 individuals (∼15.5%) that failed due to processing and QC assessment. In order to be excluded due to QC failure, individuals had to fail both the cortical ribbon and tissue segmentation visual assessments. In order to be excluded due to FS processing, the FS pipeline simply did not proceed to completion on these individuals.

When comparing Unmodified and Corrected procedures, results from a paired sample *t*-test revealed a significant increase (∼63%) in eTIV-adjusted log WM hypointensity volumes, (5824.5 ± 6378.4 mm^3^ to 15877.1 ± 17964.2 mm^3^, *p* < 0.001).

Cortical thickness analyses based on Unmodified FS revealed no significant associations with MoCA total score after accounting for age, stroke, and lacunar volumes. However, the same analyses based on the Corrected FS revealed significant clusters in the left superior parietal and left insula regions were positively associated with MoCA total score (*p*_(cwp)_ = 0.018; *p*_(cwp)_ = 0.040, respectively) (Table 3). Further analyses with the Corrected data and MoCA sub-scores using the significant clusters showed a positive association between left superior parietal thickness and the Attention sub-score.

**TABLE 3:** Cortical thickness analyses showing significant clusters with Montreal Cognitive Assessment scores corrected for multiple comparisons.

Anatomical Region	Max	Surface area of cluster (mm^2^)	Talairach (MNI305) coordinates (x,y,z)	LowCWP - HiCWP	P_cwp_
***Total MoCA score***					
Left Superior Parietal Left Insula	3.991 5.101	535.98 461.69	−25.3,−43.0,54.2 −39.5,−17.6,−10.1	0.015−0.021 0.035−0.029	0.018 0.040
***MoCA Attention***					
Left Superior Parietal	4.109	771.83	−27.9,−65.1,27.0	0.0000−0.0004	0.0002

*Abbreviations: LowCWP, Lower clusterwise *p*-value, 90% confidence interval; HiCWP, Upper clusterwise *p*-value, 90% confidence; P(cwp), clusterwise *p*-value; MoCA, Montreal Cognitive Assessment.*

## Discussion

To our knowledge, this is the first article that has proposed an improved FS processing in individuals with chronic stroke lesion and cerebral small vessel disease using an automated-hybrid approach. Most published work on improving the FS pipeline have focused on comparing the fully automated pipeline with manual interventions within FS (i.e., editing erroneous skull stripping and control points) (Li et al., 2015a; McCarthy et al., 2015; Canna et al., 2018; Klapwijk et al., 2019; Waters et al., 2019), which are labor intensive, time consuming, and may introduce user-bias. Regional cortical thickness measures obtained from participants’ MRI using FS is a useful imaging biomarker of cortical atrophy, within and between the various disease cohorts represented in ONDRI. By minimizing data loss and increasing statistical power, our correction procedures have the opportunity to develop structural neuroimaging biomarkers for early diagnosis and treatment of cerebrovascular and neurodegenerative disease. This correction procedure enabled the investigation of participants with significant atrophy and cerebrovascular lesion burden, which can present significant challenges to cortical thickness estimation, cortical and subcortical volumetrics, and other downstream processes (e.g., connectivity analyses of functional and diffusion MRI). Moreover, the correction procedures may improve the sensitivity of estimated features that may have otherwise been undetectable.

In the unmodified procedure, a failure rate of more than 60% was reported for tissue segmentation. This is in line with the concept that most segmentation difficulties reported in individuals with CVD result from inaccurate identification of tissue boundaries, which is highly dependent on the homogenous intensity of voxels in a particular brain region, especially in those with high surface area and curvature (Wang et al., 2012). Accurate and reliable skull stripping is important for cortical thickness estimation, since false positive classification of non-brain tissue (e.g., skull, dura, and pial maters) could result in poor estimation of the GM-WM border, which in turn can result in erroneous patterns of cortical thickness. Skull stripping segmentation accuracy is particularly relevant in aging and neurodegenerative populations, where brain atrophy is accompanied by increased CSF volumes and a decreased separation between GM and WM intensities (Seo et al., 2010; Walhout et al., 2015; Yau et al., 2018; Vuksanović et al., 2019; Wilson et al., 2019).

While small acute strokes may have minimal effects on tissue segmentation, large chronic cortico-subcortical stroke lesions introduce alterations to brain morphometry resulting in failed segmentation in most brain segmentation algorithms (Wang et al., 2012; Yang et al., 2016; Siegel et al., 2017; Zavaliangos-Petropulu et al., 2020). Although this issue is particularly relevant in individuals with CVD, cerebral small vessel disease and brain atrophy that are commonly observed in patients with Alzheimer’s and other related dementias present similar challenges when estimating cortical thickness.

Incorporating more accurate brain extraction and lesion masks reduced the overall failure rate from 62% down to less than 8% when the corrected procedure was applied. This improvement could be attributed to the use of multi-modal imaging sequences in the ONDRI structural neuroimaging pipeline (Ramirez et al., 2020) that produces consistent and accurate brain extraction and lesion segmentation. Although imaging markers of small vessel disease, such as WMH, appear hyperintense (bright) on PD/T2 and FLAIR MRI, these lesions appear hypointense or isointense to GM on T1, thus overlapping in intensity with normal appearing GM (Wardlaw et al., 2013). If present in confluent patches, it can result in significant inflation of GM voxel misclassification when using only T1-based segmentation approaches (Levy-Cooperman et al., 2008). Considering the significant WMH burden and atrophy in our sample, it was helpful that the FS pipeline allowed for these types of interventions. In line with this, we found a significant increase in WM hypointensities burden (∼63%) after incorporating ONDRI’s lesion segmentation to the FS pipeline. This is consistent with Magon et al. (2014) that used a lesion filling approach in multiple sclerosis to reclassify the majority of WM lesion in the WM that were erroneous classified as GM or CSF. This method improved the accuracy of tissue segmentation and classification and cortical thickness estimation. Several studies have underscored the importance of optimal lesion segmentation in various clinical population (Habes et al., 2016; Dadar et al., 2017; Sudre et al., 2017; Heinen et al., 2019; Salvadó et al., 2019), particularly in populations at risk of developing small vessel disease (Longstreth et al., 1996; Jeerakathil et al., 2004; Jongen et al., 2007; Habes et al., 2016). A recent systematic review by Frey et al. (2019) provided a comprehensive overview of the importance of WMH segmentation in large-scale MRI studies. They proposed a clear need for developing a guideline to cover the description of WMH segmentation approach, as a way of optimizing the multitude of segmentation techniques available. This is crucial, especially in medium to large sample size studies with clinical populations that donate their time to research. Furthermore, the flexibility of the FS pipeline to allow for such modification supports the individualized imaging methods used in the ONDRI study. This increases the study’s statistical power whilst including participants with challenging pathologies that otherwise might have failed when processed using the default settings, and in turn, reduces sampling bias related to the imaging method requirements (Li et al., 2015a).

Only data that underwent the FS correction demonstrated a relationship with cognition, whereby greater corrected cortical thickness in the left superior parietal cortex and in the insula was associated with higher MoCA total scores. Further analysis with MoCA sub-scores revealed that corrected cortical thickness in the left superior parietal cortex was associated in particular with higher Attention sub-scores. Several studies have reported a significant association between cortical thickness and cognitive function in participants with SVD and other diseases associated with vascular risk factors (Righart et al., 2013; Fujishima et al., 2014; Jung et al., 2014; Tuladhar et al., 2015; Tchistiakova and MacIntosh, 2016). Across these studies the effect of cortical thickness varies, with some reporting relationships with executive function, processing speed, memory (Righart et al., 2013; Tchistiakova et al., 2014), whilst others reporting relationships with memory and attention (Fujishima et al., 2014; Tuladhar et al., 2015). A study by Hilal et al. (2015) demonstrated that WMH and microbleeds were associated with thinning in the temporal and insular regions and associated multi-domain cognitive dysfunction. The insula is an important structure with extensive connections to cortical and subcortical regions, and is involved in various processes, such as empathy, emotion, body sensation, and other aspects of social cognition (Craig, 2009; Couto et al., 2013). Thus, insular atrophy as a result of stroke could lead to significant cognitive dysfunction and socioemotional deficits in participants with cerebral small vessel disease and other comorbid neurodegenerative diseases (Ibañez et al., 2010; Limongi et al., 2014; Moon et al., 2014). Further, the observed association between superior parietal thickness and the Attention sub-score is consistent with recent work showing that smoking-related superior parietal thinning was associated with decreased global cognition, as well as decreased visuospatial and attentional functioning (Neth et al., 2020). This is in line with the concept that better vascular health is associated with increased superior parietal thickness in neurodegenerative diseases (Fortea et al., 2014; Johnson et al., 2014; Vemuri et al., 2018a), suggesting a compensatory response to early brain pathological changes (Vemuri et al., 2018b). Future analyses using our method will investigate the associations between vascular risk factors and cortical thickness in predicting cognitive decline in neurodegenerative diseases with comorbid cerebral small vessel disease.

The ability to decrease the failure rate was the key strength of this work. Although our correction procedures were derived from ONDRI’s imaging pipeline, similar correction procedures that can account for vascular lesions and brain atrophy could be applied in other studies using FS (or any number of cortical thickness estimation tools) (Magon et al., 2014) to study challenging clinical populations (Dalca et al., 2014; Dadar et al., 2017; Subbanna et al., 2019; Ntiri et al., 2020). Hence, the decision to validate and apply this method to individuals with CVD presenting with a range of various combined pathologies including focal and global atrophy, large and small cortico-subcortical chronic stroke lesions, diffuse and focal WMH, lacunar infarcts, cortical microinfarcts, and enlarged PVS (Gorelick et al., 2011; Wardlaw et al., 2013). This combination of brain pathologies brings a unique set of potential challenges for cortical thickness estimation.

The findings reported here should also be considered in light of several limitations. The cross-sectional analysis of this project limits our ability to examine the robustness of our method longitudinally. As ONDRI is a longitudinal study, future work will implement our method at several follow-up time points, within and between all disease cohorts, providing a unique opportunity to investigate relationships between cortical thickness and other neurodegenerative biomarkers for predicting disease progression. As outlined in numerous studies, cortico-subcortical stroke lesions and other neurovascular lesions associated with cerebral small vessel disease are optimally visualized with multi-sequence imaging (minimally with a 3DT1 and T2/FLAIR), in cases where only T1 is available, manual intervention may be the most practical choice for cortical thickness estimation. Another benefit to the FS correction is its potential to facilitate better understanding of brain-behavior relationships by maximizing power since it prevents the loss of our valuable study participants due to poor segmentation and processing failure. As demonstrated, only the corrected cortical estimations correlated with a measure of global cognitive status, which was likely due to the increase in sample size. Future work will examine cross-sectional and longitudinal relationships between cortical thickness, vascular risk factors, neurodegeneration, and associations with more comprehensive neuropsychological testing (McLaughlin et al., 2020).

## Conclusion

Accurate topographic measurement of the cortical thickness may benefit early detection and treatment of dementia due to cerebrovascular and neurodegenerative disease. Our results suggest that the integration of the individualized accounting of brain atrophy and neurovascular lesions can significantly improve segmentation results, reduce failure rates to minimize biased samples, and maximize power to examine brain-behavior relationships. Although it is important to acknowledge that the availability of optimized neuroimaging acquisitions may be a limiting factor for implementing this correction procedure, this proof of concept study opens an opportunity for stroke and CVD researchers to potentially examine regional GM thinning in patients who previously could not be analyzed using FS’s cortical thickness tool. Most importantly, these correction efforts invested to reduce data loss and inaccuracies, recognize the significant time and effort our patients have donated to participate in the ONDRI research study. With additional funding, we seek to validate and implement this method in the Canadian Consortium on Neurodegeneration in Aging (CCNA) and other future datasets examining patients with cerebral small vessel disease, focal atrophy, and chronic stroke.

## Members of the ONDRI Investigators

Michael Strong, Peter Kleinstiver, Natalie Rashkovan, Susan Bronskil, Michael Borrie, Elizabeth Finger, Corinne Fischer, Andrew Frank, Morris Freedman, Sanjeev Kumar, Stephen Pasternak, Bruce Pollock, Tarek Rajji, Dallas Seitz, David Tang-Wai, Brenda Varriano, Agessandro Abrahao, Marvin Chum, Christen Shoesmith, John Turnbull, Lorne Zinman, Julia Fraser, Bill McIlroy, Ben Cornish, Karen Van Ooteghem, Frederico Faria, Manuel Montero-Odasso, Yanina Sarquis-Adamson, Alanna Black, Barry Greenberg, Wendy Hatch, Chris Hudson, Elena Leontieva, Ed Margolin, Efrem Mandelcorn, Faryan Tayyari, Sherif Defrawy, Don Brien, Ying Chen, Brian Coe, Doug Munoz, Alisia Bonnick, Leanne Casaubon, Jennifer Mandzia, Demetrios Sahlas, David Breen, David Grimes, Mandar Jog, Anthony Lang, Connie Marras, Mario Masellis, Tom Steeves, Dennis Bulman, Allison Ann Dilliott, Mahdi Ghani, Rob Hegele, John Robinson, Ekaterina Rogaeva, Sali Farhan, Hassan Haddad, Nuwan Nanayakkara, Courtney Berezuk, Sabrina Adamo, Mojdeh Zamyadi, Stephen Arnott, Malcolm Binns, Wendy Lou, Kelly Sunderland, Athena Theyers, Abiramy Uthirakumaran, Guangyong (GY) Zou, Sujeevini Sujanthan, Mojdeh Zamyadi, David Munoz, Roger A. Dixon, John Woulfe, Brian Levine, Paula McLaughlin, JB Orange, Alicia Peltsch, Angela Roberts, and Angela Troyer.

## Data Availability Statement

The datasets presented in this article are not readily available because the ONDRI data will be made publicly available through an application process on October, 2020. For more information on the ONDRI project, please visit: http://ondri.ca/. Requests to access the datasets should be directed to http://ondri.ca/.

## Ethics Statement

The studies involving human participants were reviewed and approved by ONDRI. Study participants were recruited at various health centers across Ontario, Canada: London Health Science Centre and Parkwood Institute in London; Hamilton General Hospital and McMaster Medical Centre in Hamilton; The Ottawa Civic Hospital in Ottawa; Thunder Bay Regional Health Sciences Centre in Thunder Bay; St. Michael’s Hospital, Sunnybrook Health Sciences Centre, Baycrest Health Sciences, Centre for Addiction and Mental Health, and Toronto Western Hospital (University Health Network) in Toronto. Ethics approval was obtained from all participating institutions and performed in accordance with the Declaration of Helsinki. All participants and study partners provided informed consent. The patients/participants provided their written informed consent to participate in this study.

## Author Contributions

MO: conceptualization, data curation, formal analysis, investigation, methodology, project administration, software, validation, visualization, and writing (draft, review, and editing). JR: conceptualization, data curation, formal analysis, investigation, methodology, software, validation, visualization, writing (draft, review, and editing), and supervision. PR: data curation, formal analysis, and writing (review and editing). MH and KW: data curation, validation, visualization, and writing (review and editing). CS: data curation, project administration, and writing (review and editing). FG: data curation, formal analysis, validation, resources, and supervision. MG: writing (review and editing). DK: data curation, project administration, writing (review and editing). MT: writing (review and editing), supervision, funding acquisition. DB: data curation, software, writing (review and editing). GS: resources, writing (review and editing). AH: resources, funding acquisition. JL-D: resources, data curation, writing (review and editing). DD and SCS: resources, data curation, funding acquisition. SS: data curation, supervision. RB: data curation, resources, supervision, funding acquisition. RS: data curation, writing (review and editing), resources, supervision, funding acquisition. SB: conceptualization, methodology, supervision, writing (review and editing), resources, funding acquisition. All authors contributed to the article and approved the submitted version.

## Conflict of Interest

The authors declare that the research was conducted in the absence of any commercial or financial relationships that could be construed as a potential conflict of interest.

## Footnotes

1 http://ondri.ca
